# Photocatalysis and photochemistry in organic synthesis

**DOI:** 10.3762/bjoc.21.128

**Published:** 2025-08-18

**Authors:** Timothy Noël, Bartholomäus Pieber

**Affiliations:** 1 Van’t Hoff Institute for Molecular Sciences (HIMS), University of Amsterdam, Science Park 904, 1098 XH Amsterdam, Netherlands; 2 Institute of Science and Technology Austria (ISTA), Am Campus 1, 3400 Klosterneuburg, Austria

**Keywords:** photocatalysis, photochemistry

Soon after its first reported synthesis in 1936 [1], [Ru(bpy)_3_]Cl_2_ (bpy = 2,2'-bipyridine) and its derivatives attracted significant attention due to their photophysical properties [2–4]. These complexes can efficiently absorb visible light through a metal-to-ligand charge transfer (MLCT) transition, resulting in a long-lived charge-separated species. In this excited state, [Ru(bpy)_3_]Cl_2_ is both a more potent oxidant and reductant than in its ground state. This reactivity, in combination with the reversible redox behavior of the metal complex, enables reductive or oxidative quenching cycles in the presence of electron donors and acceptors. Furthermore, [Ru(bpy)_3_]Cl_2_ can engage in Förster and Dexter energy transfer processes, enabling the transfer of excited-state energy to molecules that do not themselves absorb visible light. This versatility is arguably the reason for the tremendous impact of [Ru(bpy)_3_]Cl_2_ on several research areas, including solar energy conversion [5], optosensing [6], photodynamic therapy [7–8] and bioimaging [9].

Scattered examples of [Ru(bpy)_3_]Cl_2_ being used as a photocatalyst for visible-light-driven organic synthesis appeared in the scientific literature as early as the 1970s [10]. However, these remained largely isolated cases – more mechanistic curiosities than general synthetic platforms.

The narrative shifted significantly in the late 2000s, when interest in early pioneering work on photocatalysis was revisited, and systematic investigations began to demonstrate its broad applicability [11]. This renewed momentum was driven by contributions from numerous groups across the field. At the same time, advances in technology – particularly the accessibility of various light sources (most notably LEDs) – made photochemical transformations much more practical to implement. Two decades later, photocatalysis and photochemistry remain among the most studied topics in modern organic synthesis. Nowadays, chemists can choose from a wide range of organometallic [12–13], organic [14–15], or heterogeneous photocatalysts [16–17] to trigger visible-light photoredox catalysis, and this arsenal of catalysts is constantly expanding. In this thematic issue, the Dell’Amico group describes the development of a new class of organic donor–acceptor photocatalysts that show promising activity for several transformations [18]. Additionally, Hoffmann and co-workers contributed a Review article discussing photocatalysts capable of harnessing low-energy red light to trigger chemical reactions [19].

In addition to photoredox catalysis, several mechanistic platforms that leverage light – such as the use of electron donor–acceptor complexes [20], proton-coupled electron transfer [21], hydrogen atom transfer [22], halogen atom transfer [23], and energy transfer catalysis [24–25] – have been established as powerful additions to the arsenal of photon-driven reactions. Three articles in this thematic issue exemplify this: The Molloy group developed an intramolecular [2 + 2]-cycloaddition of alkenylboronic esters using energy transfer catalysis [26]. Gualandi and co-workers leveraged a combination of photoredox and HAT catalysis to realize the intramolecular nucleophilic amidation of alkenes with β-lactams [27]. Further, Luridiana and colleagues developed a method for the alkylation of a dehydroalanine derivative using silane-mediated halogen atom transfer [28].

Dual catalytic approaches that merge photocatalysis with Lewis acid [29], organo- [30], or transition metal catalysis [31] have enabled access to bond formations that are otherwise challenging to achieve. In particular, the combination of nickel catalysis and photoredox catalysis has become one of the most studied strategies to forge carbon–carbon bonds. The groups of Soengas and Rodríguez-Solla used this strategy to develop a general method for the synthesis of enaminones [32].

Light-induced transition metal catalysis that does not require exogenous photocatalysts has emerged as a new paradigm in photochemical synthesis [33]. Here, a transition metal complex plays a dual role by harnessing photon energy to facilitate bond-breaking and bond-forming events. For example, Sipos and co-workers demonstrate in this thematic issue that visible light increases the reaction rate of palladium-catalyzed Negishi cross-couplings [34].

The integration of enabling technologies has also contributed to the success of photocatalytic organic synthesis [35]. Automated reaction platforms, high-throughput experimentation techniques, and flow chemistry are being harnessed to push the limits of light-driven reactions [36]. Terada and colleagues show in this thematic issue how flow chemistry is used to significantly improve the yield of a π-Lewis acidic metal-catalyzed cyclization–radical addition sequence [37]. Recently, chemists have begun studying reactions that combine the advantages of photochemical methods and mechanochemistry. This thematic issue contains a Perspective article from the Capaldo laboratory that surveys these efforts and discusses future possibilities [38].

Without a doubt, the growing interest in light-mediated organic synthesis has also resulted in a renaissance of radical chemistry. Once regarded as “[…] messy, unpredictable, unpromising and essentially mysterious” [39], radical-based methods have become central to modern organic chemistry, spanning applications in the life sciences. The Perspective article from the Molander group on the photocatalyzed elaboration of antibody-based bioconjugates underscores this impact [40].

This thematic issue celebrates the profound impact of light-mediated synthesis across fundamental, methodological, and applied domains. As guest editors, we are deeply grateful to all contributors and thank all referees for their careful evaluations, which helped maintain the scientific rigor of this collection. We hope this collection inspires further exploration and innovation in this rapidly advancing field.

Timothy Noël and Bartholomäus Pieber

Amsterdam and Klosterneuburg, August 2025

## Data Availability

Data sharing is not applicable as no new data was generated or analyzed in this study.
